# PHM Services Based on Cyber–Physical Machine Tool System

**DOI:** 10.3390/s26123885

**Published:** 2026-06-18

**Authors:** Chuting Wang, Ruijuan Xue, Xuesong Mei, Zuguang Huang

**Affiliations:** 1 General Technology Key Laboratory of High-End CNC Machine Tools, Beijing 100102, China; wangchuting@sict.ac.cn (C.W.);; 2 School of Mechanical Engineering, Xi’an Jiaotong University, Xi’an 713599, China; 3 Frontier Institute of Science and Technology, Xi’an Jiaotong University, Xi’an 713599, China

**Keywords:** PHM, CNC machine tool, digital twin

## Abstract

Heterogeneous fault information and a lack of real-time synchronization in CNC machine tools hinder effective Prognostics and Health Management (PHM). This paper designs and implements a digital twin-driven PHM framework for machine tools that integrates a unified machine-tool fault information dictionary and a mechanism-data dual-driven diagnostic model (ResNet-TCN). A cyber–physical platform was developed using OPC UA and RESTful APIs to ensure real-time data synchronization. Experiments on the PHM 2010 dataset demonstrate that the proposed ResNet-TCN model achieves a root mean square error (RMSE) of 5.46 μm for tool wear prediction. Its performance surpasses that of traditional LSTM models, and the proposed framework effectively eliminates information silos, providing a responsive, scalable and accurate PHM solution for smart manufacturing.

## 1. Introduction

With the advent of the Internet of Everything (IoE) era, the rapid development of a new generation of information technology [1], such as big data, cloud computing and the Internet of Things, has further promoted the integration of information technology and CNC technology [2], and the CNC system has stepped into a new stage of digitization, intelligence and servitization. Digital twins are regarded as an enabling technology for the intelligent transformation of CNC machine tools [3]. The physical world and the cyber world will be integrated. through the construction of the digital twin model of the machine tool (MTDT) [4], real-time monitoring and feedback control of the machine tool state and machining process, and the integration of a variety of intelligent applications into the CNC system.

CNC machine tools are located in a harsh processing environment, complicated working conditions, complex fault information, and high probability of failure [5]. Thus, the fault diagnosis and health management (PHM) of machine tools has become one of the most important intelligent services for users. PHM prompts operators through the fault alarm information provided by the CNC system early and quickly solves the obstacles of CNC machine tools to ensure smooth production, and it is widely used in the tool wear of CNC machine tools as well as condition monitoring, health management, fault diagnosis and spindle prediction.

Despite progress in the field of PHM, a key research gap remains: existing digital twin driven-PHM studies either focus on isolated data-driven algorithms that lack standardized fault semantics, resulting in an inability to handle heterogeneous fault codes from different machine tool manufacturers [6]; or they propose complex artificial intelligence models without explaining how these models can be integrated with real-time, standardized communication architectures such as OPC UA. Consequently, there is an urgent need to establish a unified framework that combines the modelling of standardized fault information with high-real-time digital twin synchronization capabilities.

In order to solve the above problems, this paper realizes PHM services for machine tools via digital twin technology. By constructing a faults MTDT based on the historical information of equipment, this paper realizes a unified representation of CNC machine tool fault information that is able to analyze changes in the temperature, load and position of environment, machining objects and machine tool equipment through sensor acquisition and computer technology. Through establishing a fault information management system and using intelligent algorithms such as machine learning to monitor and statistically analyze the collected real-time data of CNC machine tools in the workshop, this paper provides PHM for machine tools. Finally, by developing a visualization platform, this paper help users to grasp the specific situation of machine tools and workshops in a centralized, timely and effective manner.

The remainder of this paper is organized as follows: Section 2 describes specific methods for implementing PHM for machine tools using digital twins; Section 3 provides a detailed introduction to the machine tool digital twin model established for the purpose of PHM; Section 4 sets up the experimental platform and demonstrates the client-side interface for machine tool PHM; Section 5 summarizes the research findings of this paper and outlines future research directions.

## 2. Literature Review

PHM [7] is a multidisciplinary integrated technology that employs sensors to monitor the real-time operational states and characteristic data of products/systems. Utilizing artificial intelligence algorithms, it monitors and predicts the health status of products/systems, enabling pre-failure prediction and alerting operators and managers. Having progressed through stages of reactive post-failure maintenance and periodic scheduled maintenance, PHM has now entered the era of proactive predictive maintenance [8]. Fault prediction can be understood as PHM’s capability to diagnose faults based on a machine tool’s future health status. By collecting operational parameters, it enables real-time monitoring of the machine tool’s operational state, assesses its health condition, and performs fault diagnosis and lifespan prediction using historical data and artificial intelligence algorithms. This facilitates the elimination of potential faults and maintenance before failures occur. In most instances, PHM simultaneously avoids unnecessary component replacements or downtime maintenance, thereby reducing production costs while preventing major failures or accidents caused by untimely maintenance. This approach eliminates unpredictable economic losses and potential safety hazards, resulting in higher overall maintenance efficiency. Current PHM approaches primarily fall into three categories: model-based, data-driven, and statistical probability-based, as shown in Table 1.

Model-based PHM constructs specific physical and mathematical models based on the internal working mechanisms of complex equipment. Subsequently, by integrating corresponding physical laws, it analyzes the operational state of the equipment to reflect its performance degradation patterns [9]. Through setting boundary conditions for model simulation and solution, it predicts equipment failure scenarios and performance degradation trends, thereby formulating maintenance plans. Hector et al. [10] proposed a model predictive control scheme embedding a stiffness degradation model within the prediction algorithm to forecast the equipment’s remaining useful life (RUL). Lu et al. [11] proposed a deep spatio-temporal network based on interactive attention. This network integrates multi-sensor signals. Although it is capable of simultaneously extracting long-term dependencies and local spatial information from raw multi-source data of fuse vibration waveforms and time-varying operational signals, it still relies on time windows of fixed length.

Data-driven PHM necessitates the collection of condition monitoring data from in-service equipment. Whilst analysis need not be conducted on corresponding physical models, it requires integrating a physical understanding of the system to perform the appropriate monitoring data analysis. Current mainstream data-driven approaches predominantly employ machine learning algorithms to train diagnostic models [12]. Zhong et al. [13] proposed an RUL prediction method combining adaptive activation functions with attention mechanisms, which is capable of simultaneously extracting long-term and short-term information while constraining all eigenvalues within a defined range. Mandelli et al. [14] proposed a reliability method for reserve capacity based on conditional data, evaluating equipment operational status through diagnostic/prognostic assessments and monitoring data. Cinus et al. [15] introduced a decision support system utilizing artificial neural networks (ANNs) to process sensor data and key performance indicators (KPIs), integrating maintenance operations into weekly production schedules.

PHM based on reliability statistical probability typically employs the statistical characteristics of historical failure data for forecasting. By utilizing extensive, long-term reliability statistics or reliability test data, corresponding statistical probability functions are constructed to enable prediction [16]. Christen et al. [17] proposed a predictive theory for equipment reliability evolution within the Fokker–Planck framework, which is based on the mathematical equivalence between FPE and the Langevin equation. Gazi et al. [18] introduced a user-friendly decision table method integrating Weibull analysis, machine learning techniques, and system state as maintenance budget levels, enabling a flexible selection of maintenance plans. Abdulaziz et al. [19] proposed a system-level criticality analysis method based on fuzzy ANP. This method assigns criticality scores to 11 components of a CNC lathe based on cost, complexity, sustainability, functional dependency, and safety impact, thereby formulating predictive maintenance strategies.

**Table 1:** Comparison of model-based, data-driven, and statistical PHM approaches.

Category	Advantages	Limitations	Representative Studies
Model-based	physically interpretable;	requires accurate system model;	[6,16]
	no need for large failure data	computationally expensive for complex systems	
Data-driven	handles complex, nonlinear systems;	requires large labeled datasets;	[10,12]
	scalable with data	black-box nature; sensitive to data quality	
Statistical	works with limited data;	assumes stationary failure distributions;	[13,17]
	provides confidence intervals	not adaptive to changing conditions	

Current research indicates that the importance of predictive maintenance for manufacturing and other industries is widely recognized. It is also crucial for ensuring equipment remains efficient and sustainably operational. However, owing to the limitations of predictive maintenance, the primary maintenance models currently adopted by enterprises remain reactive maintenance and large-scale redundant preventive maintenance. Concurrently, the emergence of digital twin technology presents fresh opportunities for traditional industries. Since 2004, numerous major corporations within the sector have begun leveraging digital twin technology, undertaking many beneficial trials in product design, manufacturing, and service provision [20]. The integration of digital twins with predictive maintenance has also emerged as a new trend in recent years.

Tao et al. [21] constructed a five-dimensional digital twin model for complex equipment, proposing a novel DT-driven PHM methodology. This approach effectively leverages the interactive mechanism of digital twins to mitigate environmental interference, potential equipment failures, and model deficiencies, ensuring PHM accuracy through physically–virtually integrated data. B.D. Deebak et al. [22] proposed a digital twin-assisted fault diagnosis method based on deep transfer learning to analyze machining tool operating conditions. Integrating a K-type thermocouple and cloud data acquisition system onto the tool holder’s WiFi module enhanced precision, optimizing milling and drilling operations for cutting tools. Luo et al. [23] proposed a hybrid predictive maintenance method for CNC machine tools driven by digital twin models and data. By establishing a multi-domain digital twin model reflecting actual operating conditions and utilizing multiple sensor types to collect data, they developed a data-driven remaining useful life prediction model. Through particle filtering algorithms integrating system observations with theoretically derived values, they achieved predictive life assessment for cutting tools. Yu et al. [24] constructed a digital twin model based on non-parametric Bayesian networks to represent the dynamic degradation process of health status and propagate cognitive uncertainty. They proposed a real-time model update strategy combining improved Gaussian particle filtering (GPF) and a Dirichlet process mixture model (DPMM) to enhance model adaptability. The feasibility of the digital twin approach and the effectiveness of the non-parametric Bayesian network were validated through optoelectronic system experiments. Werner et al. [25] proposed a methodology for predictive maintenance strategies, addressing the acquisition, processing, and analysis of historical field data alongside the generation of corresponding simulation data. By integrating the digital twin concept for production machinery, they demonstrated the interaction between measured and estimated data alongside simulation-generated data. Digital twins can furnish outcomes to refine data-driven predictive models, thereby improving RUL estimation. Wihan Booyse et al. [26] proposed a general framework for prognostic and health monitoring that can be rapidly deployed across heterogeneous asset fleets. This framework employs deep generative models to construct digital twin models, learning the distribution of health data directly from operational data at the outset of a device’s lifecycle. This approach avoids relying on historical failure data to estimate device health, enabling early fault prediction and the tracking of device degradation.

Existing research indicates that traditional predictive maintenance functions solely as a computational tool for analyzing equipment status, forecasting failures, and supporting decision making. It fails to account for the complex interactions and evolutionary mechanisms within a machine’s structural and physical properties nor does it consider real-time parameter variations during data processing. Consequently, the resulting data analysis may not accurately reflect the dynamic physical state of the machine tool, thereby compromising the precision of equipment health assessments and predictions. Although academic and industrial circles have conducted research in areas such as digital twin technology, existing studies on predictive maintenance for CNC machine tools remain limited. Most research is confined to static models or single-domain construction, failing to comprehensively characterize fault information under complex operating conditions. GE’s (General Electric Company, Boston, MA, USA) “Predix” platform [27] provides tools for creating digital twins and is used to perform data analysis and monitoring. Siemens (SIEMENS AG, Munich, Germany) has developed the MindSphere platform [28], which supports a wide range of communication protocols including MQTT, OPC UA, Modbus, S7, HTTP and HTTPS, and it is capable of analyzing and making decisions based on data streams numbering in the billions. However, as these platforms are all deployed on cloud servers, they struggle to meet the requirements for high real-time data processing and data privacy that arise during the actual operation of CNC machine tools.

This paper introduces a digital twin-driven PHM methodology for machine tools to tackle these issues, offering novel approaches and pathways for fault diagnosis and health management in existing machine tools. Unlike communication protocols such as OPC UA and MTConnect, which focus solely on data acquisition, cloud platforms prioritize the storage of vast amounts of data rather than real-time performance. This framework utilizes the OPC UA protocol for data extraction and to enable real-time bidirectional control feedback whilst employing a standardized fault dictionary to address the challenge of extracting semantic information from multi-source, heterogeneous data.

## 3. Implementation Methods for PHM of Machine Tools Driven by Digital Twins

**Figure 1:**
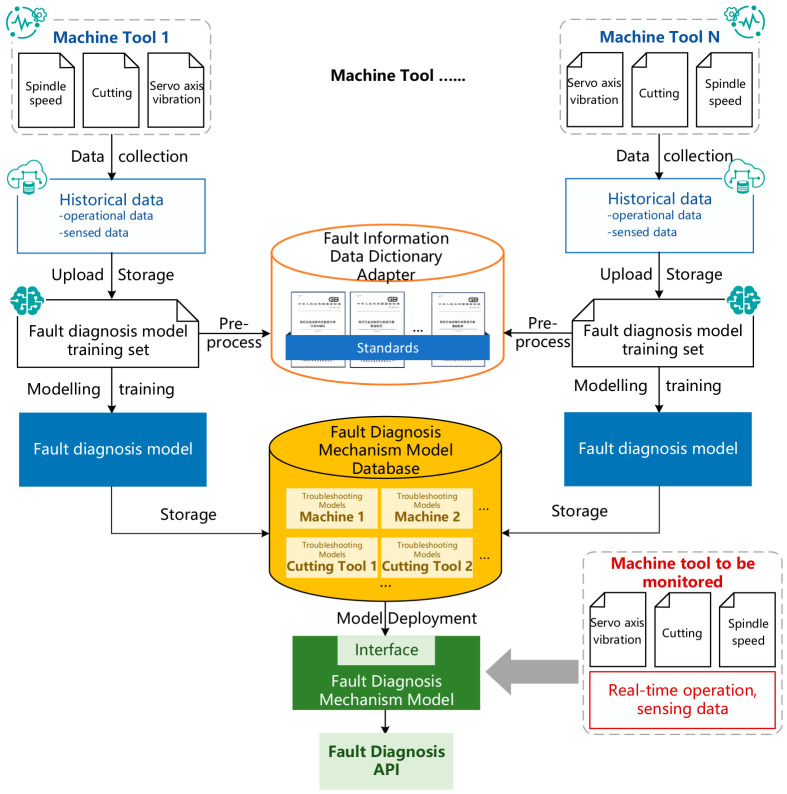
CNC machine tool fault information diagnosis implementation method.

The method for implementing the PHM of machine tools via digital twins is illustrated in Figure 1. The core concept is to provide the output from the machine tool fault information data dictionary and to transmit historical parameters and sensor data from the machine tool’s operation to the fault diagnosis module, thereby enabling fault diagnosis and indexing within the fault diagnosis model. First, status information is gathered from sensors (such as those measuring force and acceleration), PLCs, drives and other components of the machine tool. Leveraging the robust cross-functional integration capabilities of the digital twin, all machine tools within the production workshop are connected through data acquisition [29]. This enables the capture of relevant real-time status data, which is then encapsulated in a standardized format and transmitted to the data storage module. This procedure meets the criteria for evaluating the health status of the machine tools.

In order to meet the needs of rapid collection, CNC equipment needs to be connected to the Internet. For machine tools with network sockets, communication is relatively simple. However, in the actual production environment, due to differences in production and processing processes and sequential processes, shop floor CNC machine types are often difficult to unify. Different manufacturers produce different kinds of CNC systems corresponding to different communication methods. Heterogeneous protocol standards produce different communication interfaces. Connecting all CNC machines to a network requires developing upper-layer applications directly on vendor-specific native interfaces. This approach is difficult to implement. It also hinders unified data acquisition. Therefore, a unified and scalable data model of the CNC system is needed to transform all the collected information. At the same time, in order to meet the high real-time nature of the collection platform, the data information of multiple CNC equipment needs to be collected concurrently.

After completing the data acquisition, for the secondary development of CNC systems, the machine tool fault information data dictionary is built into the CNC system, and the fault code information is output by the control system with the built-in CNC machine tool fault information data dictionary. For CNC machine tools that do not support secondary development of the CNC system, an external adapter type implementation method can be used, and the fault code information is output by the equipped external adapter. The relevant fault codes are queried from the CNC machine tool fault information data dictionary database according to the fault information index to generate a fault diagnosis training set for the training of the fault diagnosis model. The fault diagnosis training set comprises 120,000 samples, covering four major categories of faults: controllers, servo motors, functional components and machine tool accessories. The issue of class imbalance was addressed using Synthetic Minority Over-sampling (SMOTE). Pre-processing was carried out using wavelet denoising and the Fast Fourier Transform (FFT) to extract frequency-domain features. The trained fault diagnosis model is stored in the fault diagnosis mechanism model database, and the mechanism model of fault diagnosis is deployed through the communication interface. Among them, the fault information dictionary for CNC equipment with built-in implementation can be accessed directly through the network, while the fault information dictionary for CNC machine tools with external adapters needs to be accessed through the adapter to achieve network access.

Finally, fault diagnosis is performed for the real-time data of the machine tool to be monitored, and fault diagnosis service is provided to users through RESTful API. OPC UA is used for real-time data transmission, whilst the monitoring data push service utilizes WebSockets to maintain a consistent refresh rate of 10 Hz. In the workshop site, after the fault inquiry system queries the CNC machine tool fault, it follows the fault information data format by querying the fault classification/code library and outputs the fault information through existing or following the developed national and international communication standards. The data listener obtains the data of remote interactions and fault information between each CNC system and upstream management system and then transmits it to the upper analysis software for fault information format and content analysis. After receiving the remote interaction data from the data listener, the analysis software parses and displays the data according to the communication standards used by the CNC system, and then it assists the machine operator in maintenance or repair.

## 4. Digital Twin Model of Machine Tool

Digital twins form the core of intelligent application services. In our previous work [3], the digital twin model of a machine tool is divided into two parts: an information model and a mechanism model. Among them, the information model comprehensively portrays the logical structure of the machine tool in a hierarchical manner, reflecting the coupling relationships among components and systems [30], grouping all available data items related to a specific component, and providing standardized and extensible data management [31] for the effective manipulation and utilization of real-time manufacturing data for further analysis. Mechanism models are new AI + mechanism models constructed by introducing artificial intelligence algorithms based on traditional mathematical mechanism models such as expert experience and domain knowledge [31], which are capable of acquiring specific data from the information model to enable real-time monitoring and prediction as well as autonomous optimization, provide decision support, and support the application services of machine tools.

**Figure 2:**
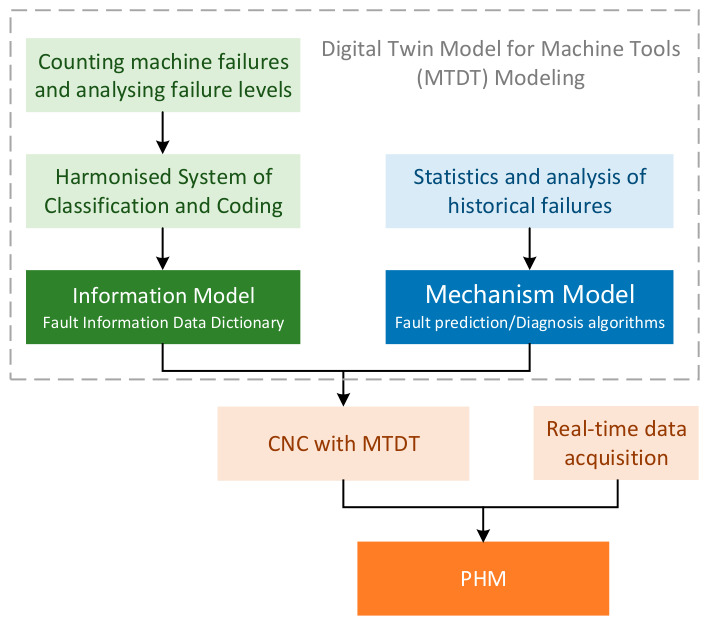
Modeling and working process of MTDT.

The modelling and working process of the digital twin model of machine tool PHM is shown in Figure 2. Firstly, the existing fault classification and related fault codes of domestic and foreign manufacturers are investigated, and the faults are classified according to the parts of the CNC machine tool where the faults occur so that uniform fault classification and coding is carried out for CNC machine tools from different manufacturers. Secondly, we establish a CNC machine tool fault information data dictionary, i.e., an information model, to achieve the unification of machine tool fault information coding and expression format. Asa result, the fault information data at the equipment layer can be directly connected to the workshop layer, factory layer and remote operation and maintenance system, enabling users to conveniently and quickly query the relevant fault information of the CNC machine tools. Then, the mechanism model, i.e., fault diagnosis and prediction algorithm, is established based on the historical fault analysis of the machine tool. Finally, the workshop fault information management system is built on the CNC system with the built-in fault information data dictionary, which monitors and statistically analyzes in real time the operation status of CNC machine tools in the workshop, the machining progress, the equipment operation efficiency and other data as well as provides PHM services through API.

### 4.1. Information Model

The information model in this paper, which can also be called the CNC machine tool fault information data dictionary, consists of two parts: the machine tool fault code and the fault card, where the fault code is the code that can uniquely identify a specific fault that the CNC machine tool has. This paper establishes the machine tool fault information model, specifies the content, classification and coding of machine tool faults, establishes the query index, and specifies the sources and processing methods of faults in the fault information model.

**Figure 3:**
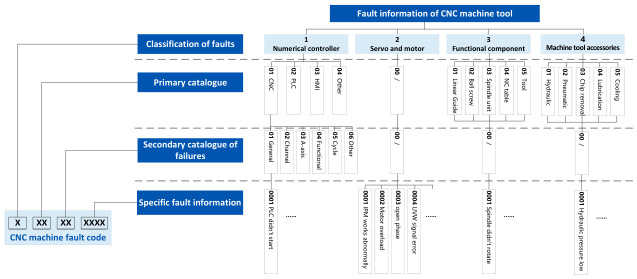
The information model of the CNC machine tool system fault.

The CNC machine fault code can uniquely identify a specific fault that the CNC machine has. The fault code consists of nine bits, each of which specifies a different meaning, as shown in Figure 3. Bit 1 shows the classification of the fault. Bits 2–3 show the primary catalogue of faults; i.e., under the fault system classification of the CNC equipment fault information, each fault set is split into a number of fault objects defining the specific fault components. Bits 4–5 show the secondary catalogue of faults, i.e., defining the faults of the next level of catalogue of faulty components under the primary catalogue. The primary and secondary catalogue of faults parts are coded from 01. Faults that cannot be categorized are entered in the “Other” category. Bits 6–9 indicate the specific fault information, which is coded from 0001.

**Figure 4:**
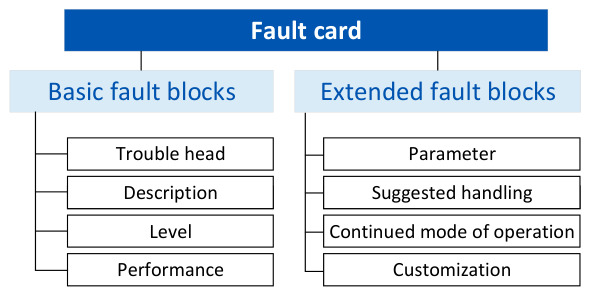
Fault information object model.

The fault card is also called the CNC machine tool fault information format, which mainly regulates the composition of fault information, layout form constraints, text block structure, text block constraints and the corresponding name and logical meaning. As shown in Figure 4, the fault card includes basic fault blocks and extended fault blocks in two separate parts. The basic fault block is the text block that every fault should contain, while extended fault blocks can be selectively included according to the specific fault information category. The logical meaning and role of each text block are shown in Table 2.

**Figure 5:**
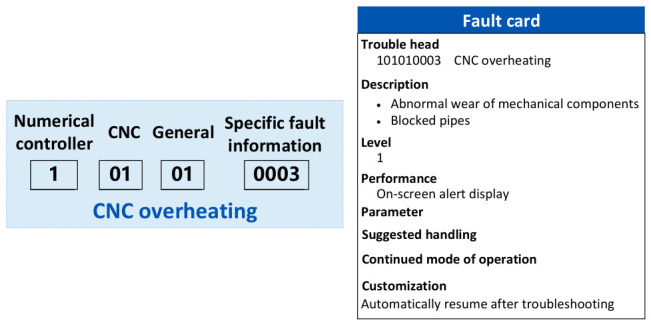
Examples of fault coding and fault card.

For the ‘CNC overheating’ fault, different CNC system manufacturers provide varying fault codes; for example, the FANUC CNC system displays the fault alarm code ‘OH0700’, Siemens uses ‘2110’, and Mitsubishi uses ‘0113’. As a result, workshop managers are unable to quickly identify the fault information. By using the data dictionary proposed in this paper, these codes can be uniformly mapped to standardized fault codes. To aid understanding, Figure 5 provides examples of fault coding in industrial applications and the resulting fault card.

### 4.2. Mechanism Model

CNC machine tools are composed of different subsystems and components, each of which has its own mechanism model for fault diagnosis and prediction. Since the mechanism model of machine tool PHM covers a wide range, this paper introduces the mechanism model of tool wear diagnosis and prediction application service as an example only.

In the machining process, tool wear accompanies the entire machining cycle, and failure to provide a timely warning of tool failure during machining will affect the quality of machining and even pose a great threat to machining safety [32]. However, there are many possible disturbing factors for tool wear, and each tool has a specific wear curve. Due to the tool bite and cutting fluid, it is not feasible to directly measure the tool wear condition during machining. In addition, since there is no accurate physically-based tool wear assessment model, conventional tool wear diagnosis is usually based on workers’ experience and statistics, which is an approach that may lead to untimely or excessive maintenance.

To address the above problems, Sun et al. [33] proposed the use of residual networks to monitor tool wear in real time. Existing studies generally agree that the deeper the network, the higher the model accuracy; however, the degradation problem often occurs along with the increase in the network depth, i.e., as the number of network layers increases, the model accuracy decreases. Residual network [34] introduces Residual Learning (RL) through a short-circuit mechanism on the basis of VGG19, which can effectively solve the deep network degradation problem while guaranteeing the network depth. The main idea of a residual network is to map into a constant shortcut connection so that the upper layer features can be passed into the lower layer network without loss, thus circumventing the weight layer.

Another focus of tool wear diagnosis and prediction is the prediction of tool wear values, i.e., the prediction of the future condition of tool wear by calculating the obtained tool wear values. This prediction mostly relies on the time-series model, and the Recurrent Neural Network (RNN) represented by LSTM and GRU is considered to be the preferred solution to the sequence model. However, in practice, the internal design of the RNN has serious problems; i.e., the network can only process one time step at a time, which prevents massively parallel processing, and at the same time, it is extremely computationally intensive, as all intermediate results must be saved until the end of the whole task. These problems are effectively solved by the emergence of the Temporal Convolutional Network (TCN). This type of network has a fully convolutional network with the same input and output lengths, and it introduces causal convolutions to avoid the omission of historical data from the prediction process, and dilated convolutions for the learning process. Convolutions enable adjusting the learning horizon and prevent the network structure from being too large. In this paper, TCN is utilized for tool wear prediction considering its advantages of having a flexible sensory domain, better control of the model’s memory size, stable gradient, low memory requirement during training, and variable input length.

Specifically, structured fault information (i.e., the fault cards) is converted into a one-hot encoded vector and concatenated with normalized sensor time-series data. This multimodal fusion provides the ResNet-TCN model with physical context, thereby accelerating convergence and enhancing the interpretability of diagnostic results.

**Figure 6:**
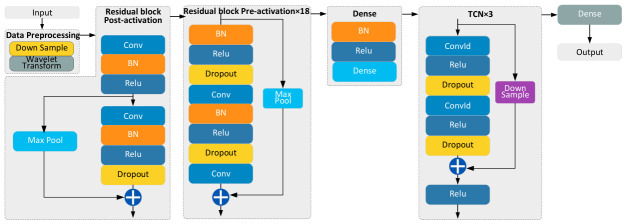
The PHM model of cutting tool.

As shown in Figure 6, the tool wear diagnosis and prediction functions are supported by a combination of residual networks and TCNs. The tool PHM model mainly consists of a data preprocessing module, one post-activation residual block and 18 pre-activation residual blocks that provide a real-time diagnosis of tool wear, and three TCN blocks that provide wear prediction functions. A tool wear diagnosis and prediction model was trained using the IEEE PHM 2010 challenge dataset [35]. A stratified sampling strategy is adopted to split the data: 60% for training, 20% for validation, and 20% for testing. LSTM, TCN, and the proposed ResNet-TCN are trained and evaluated under identical conditions with the same preprocessing. The input data consisted of a seven-dimensional dataset comprising the X, Y and *Z*-axis forces, the three-axis accelerations, and the AE signals recorded during the machining process. The hardware configuration comprised an Intel i9 11900K CPU and an NVIDIA GeForce RTX 3090 GPU with the model trained for 200 epochs. As shown in Table 3, model performance is evaluated using root mean square error (RMSE), mean absolute error (MAE), and mean absolute percentage error (MAPE).

## 5. Experimental Platform Construction

### 5.1. Experimental Platform

**Figure 7:**
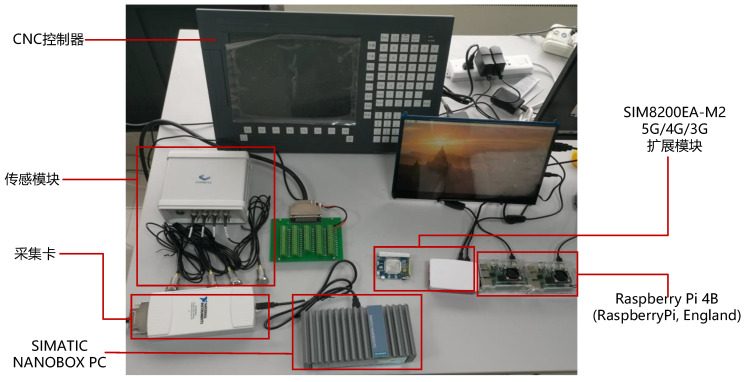
Hardware components of the machine tool PHM system.

The hardware components of the machine tool PHM system based on digital twins established in this paper are illustrated in Figure 7. On the software front, all software is integrated within the CNC controller, including an OPC UA server and machine tool PHM service module developed on the open-source LinuxCNC platform.

**Figure 8:**
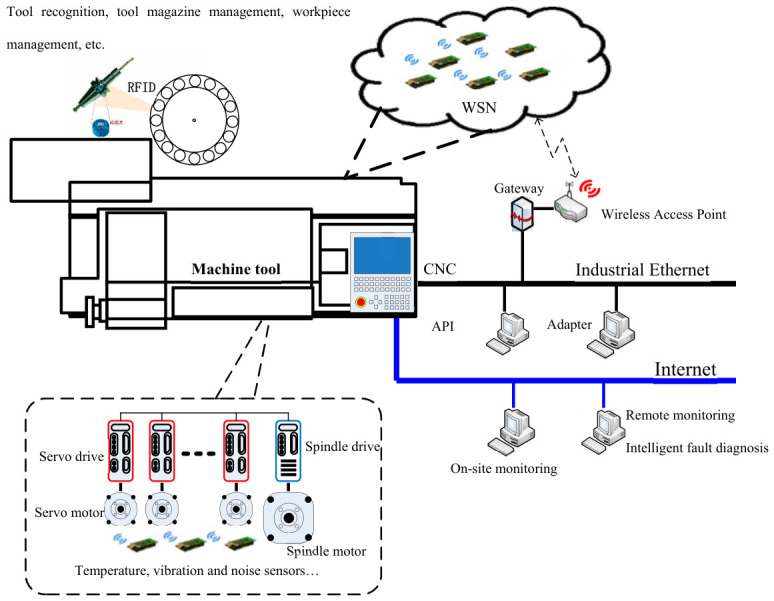
Workshop floor network structure.

To fulfill the functional requirements of PHM for machine tools driven by digital twins, networked integrated management and the control of production units is necessary. This involves establishing data communication between the control system, the data communication platform, and the execution layer of field equipment through the industrial networking of on-site devices, thereby constructing a platform for connecting heterogeneous, multi-source equipment and data acquisition. The workshop floor network architecture, as illustrated in Figure 8, adopts an open, networked structure to facilitate the networking and data acquisition of CNC machine tools on the shop floor. Data sharing is achieved through switches and industrial Ethernet.

**Figure 9:**
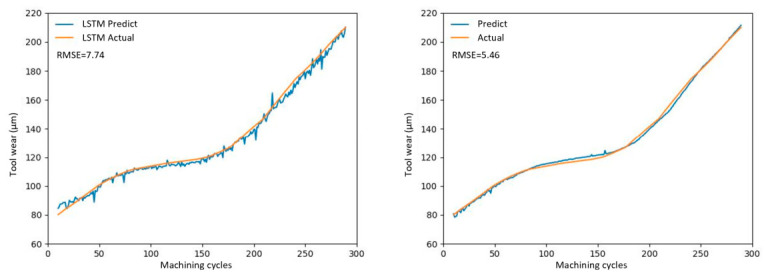
Prediction results of tool wear.

The experimental validation was carried out on three machining centers over a period of three months. The test scenarios involved milling aluminum alloy workpieces using a 10 mm carbide end mill. The cutting parameters included spindle speed (1000 to 3000 rpm) and feed rate (100 to 300 mm/min). Actual tool wear data were obtained through offline microscopic measurements taken every 30 min. The model was trained for 200 iterations with tool wear values from 15 machining operations used as input to predict tool wear after 10 machining operations. The results are shown in Figure 9, where the predicted curve closely matches the actual wear trend. The model’s loss was described using the RMSE. Compared with the LSTM (RMSE = 7.74), the algorithm proposed in Section 4.2 exhibited lower loss (RMSE = 5.46) and better fitting performance.

### 5.2. PHM Application Service

This paper employs a front-end/back-end separation architecture to develop an OPC UA-based machine tool PHM application service. The front-end utilizes Vue.js to build the user interface in conjunction with the Ant Design Vue component library; the back-end is based on the .NET platform, using C# to develop RESTful API services. The physical–cyber synchronization error was maintained below 5 ms. The DT model update frequency was set to 50 Hz with a data throughput of 2.5 MB/s per machine tool. The packet delivery reliability of the OPC UA/MQTT communication architecture reached 90%, demonstrating high scalability and reliability for workshop-level deployment.

**Figure 10:**
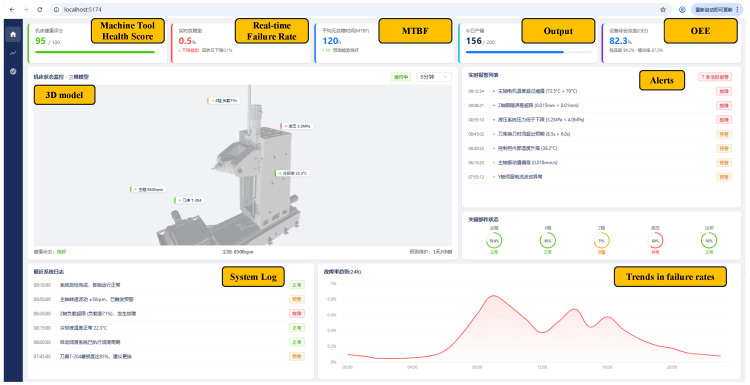
Client of machine tools PHM.

The client interface is shown in Figure 10. The top section displays an overview of the machine tool, including its health score, actual failure rate, mean time between failures (MTBF), output, and overall efficiency. The central section presents a 3D model of the machine tool using visualization with component health status indicated by different colors. The bottom section displays system logs and a 24 h trend of the failure rate. Additionally, a manual time interval adjustment function is provided, allowing the user interface to automatically refresh information at preset intervals (e.g., 10 min). The average task latency (from the occurrence of a fault to the display of an alarm on the interface) is 250 ms.

**Figure 11:**
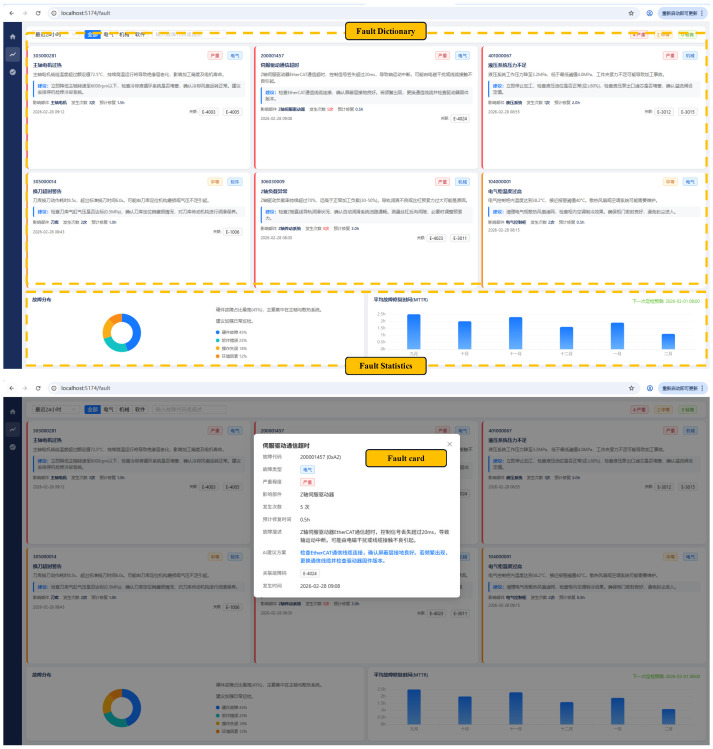
Fault diagnosis interface.

A fault diagnosis module has been designed to provide comprehensive oversight of machine tool usage. It comprises two functions: a fault dictionary and fault statistics, as shown in Figure 11. The fault dictionary allows users to view historical fault records, whilst the fault statistics function utilizes data mining and analysis to provide time-segmented queries regarding the frequency and categories of faults. It displays fault codes and the number of faults in bar charts, and it presents the average time between failures in graphical form. By utilizing recorded scheduled maintenance data, it calculates the date of the machine tool’s next scheduled inspection and displays the time remaining until the next maintenance, enabling maintenance personnel to identify recurring issues and, based on these prompts, quickly pinpoint machine tools approaching their scheduled inspection dates for maintenance. A table display function is also provided with support for exporting data to Excel.

**Figure 12:**
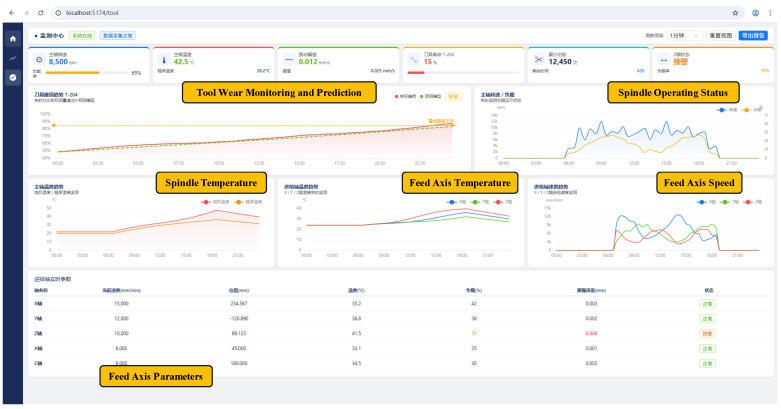
Monitoring interface.

Real-time monitoring is one of the key concerns for PHM service users. By monitoring the status of functional components such as cutting tools, spindles and feed axes in real time and with precision, it ensures the smooth running of production whilst reducing cost losses and improving production efficiency and quality. The monitoring interface is shown in Figure 12; at the top of the interface are status tabs for each component, and users can manually adjust the time interval to control the refresh rate. The system also supports report export functionality. The following charts illustrate the spindle speed/load, spindle temperature trends, feed axis temperature and speed amongst other parameters. In particular, the warning thresholds are determined in accordance with the ISO 10816 [35] and ISO 230 [36] series of standards.

**Figure 13:**
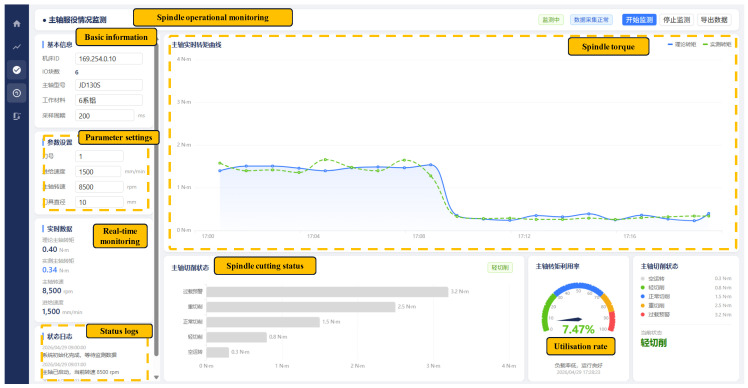
Spindle operational monitoring interface.

The spindle operational monitoring interface is shown in Figure 13. The left-hand side of the interface displays basic information such as the spindle model and workpiece material, allows users to set parameters such as emergency speed and spindle speed, and provides quick access to real-time monitoring data and status logs. The right-hand side of the interface displays spindle torque in the form of a line graph with blue representing theoretical torque and green representing measured torque. The spindle cutting status is displayed in the form of a bar chart, showing the spindle torque utilization rate, with different colors indicating the cutting status.

**Figure 14:**
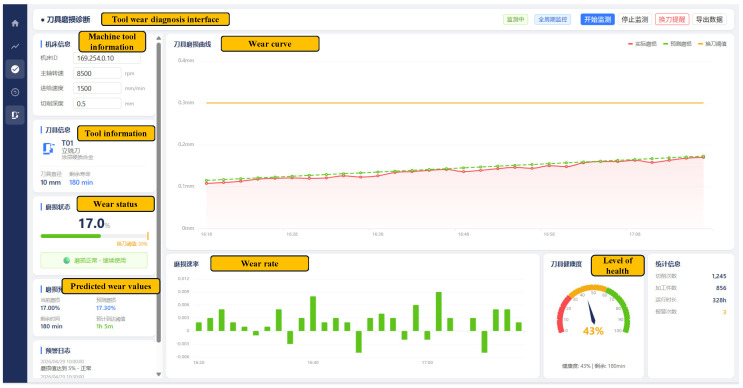
Tool wear diagnosis interface.

The tool wear diagnosis interface is shown in Figure 14. By integrating the tool wear module into the mechanistic model described in Section 4.2 of this paper, the tool’s health status is monitored in real time throughout its entire lifecycle. The left-hand side of the interface displays the machine tool information, tool information, wear status and predicted wear values, amongst other details. On the right-hand side of the interface, users can view actual and predicted tool wear values in real time via a line chart, enabling them to promptly identify tool breakage, chipping and wear. In the chart, the red line represents actual wear values, whilst the green line represents predicted wear values. Tool replacement thresholds are preset based on historical data; when wear values reach the threshold, a replacement alert is triggered, and the tool’s health status is indicated by different colors to prevent workpiece scrap resulting from untimely tool replacement.

Quantitative evaluation of the communication layer is critical for industrial applications. Therefore, stress tests were conducted to measure the latency, transmission stability, and synchronization error between the physical CNC machine and the virtual twin. Under a typical workshop network environment, the average end-to-end latency via WebSockets is 18.5 ms. During a 24 h continuous transmission test, the packet loss rate remains below 0.05%. The synchronization error, defined as the time gap between physical state changes and digital twin platform updates, is maintained at approximately 45 ms with a 10 Hz refresh rate. Although data latency affects PHM decisions, delays below 50 ms are negligible for tool condition monitoring in our framework, as actual tool wear evolves over minutes or hours.

Although this paper provides a portable and scalable digital twin reference paradigm for machine tool PHM, contributing to the advancement of autonomous health management in discrete manufacturing workshops, certain limitations remain. Factors such as complex electromagnetic interference in real factories, dynamic network topologies, and long-term sensor aging are not considered. Future work will deploy the proposed digital twin framework in a real manufacturing workshop for long-term validation. Edge computing will be introduced to further reduce latency, and the fault information model will be extended to cover a broader range of machine tool components.

## 6. Conclusions

Digital twins emerged in the era of intelligent manufacturing, which is characterized by the deep integration of next-generation information technologies with the manufacturing sector. The transformation, upgrading, and development of manufacturing have rendered predictive maintenance increasingly vital. This paper proposes a digital twin-driven PHM methodology for machine tools. By constructing information models and mechanism models tailored to machine tool failures, it achieves a comprehensive characterization and prediction of such failures. An experimental platform was established to validate the feasibility of the proposed approach. The system has been primarily validated in a laboratory environment. Achieving fully autonomous health management and large-scale industrial deployment requires long-term testing on multiple physical devices under complex industrial conditions. Future research will integrate edge computing to enable lower-latency processing. The platform robustness will be validated in real manufacturing workshops to address these limitations.

## Figures and Tables

**Table 2:** Text block logic meaning and function.

Logic Meaning	Function
Trouble head	Locate specific faults in CNC equipment, including fault numbers and fault names
Description	Reveals the cause of the fault
Level	Describes the failure level; failure level 0 does not affect the processing, failure level 1 affects the processing but is non-destructive, and failure level 2 affects the processing and is destructive
Performance	The system performance when a fault occurs can be divided into intrinsic and extrinsic performance. Intrinsic performance includes fault indicators, etc., while extrinsic performance includes the vibration and noise of equipment, etc.
Parameter	Fault parameter accompanying the fault
Suggested handling	Failure suggested handling
Continued mode of operation	The way the system continues to operate after troubleshooting, i.e., whether the system needs to be powered down and restarted or whether it runs spontaneously after troubleshooting.
Customization	Customized additional text blocks

**Table 3:** Performance of different models.

Models	RMSE	MAE	MAPE
LSTM	7.74	3.52	7.21
TCN	6.12	2.67	5.33
Res-TCN	5.46	2.18	4.73

## Data Availability

The original data presented in the study are openly available in 2010 PHM Society Conference Data Challenge at [34].

## References

[1] Reshmi T.R., Azath M. (2021). Improved self-healing technique for 5G networks using predictive analysis. Peer-to-Peer Netw. Appl..

[2] Xiao W., Zhang K., Wang S., Xiao J., Xing H., Li R., Zhao G. (2024). STEP-NC enabled edge–cloud collaborative manufacturing system for compliant CNC machining. J. Manuf. Syst..

[3] Wang C., Guo R., Yu H., Hu Y., Liu C., Deng C. (2023). Task offloading in cloud-edge collaboration-based cyber physical machine tool. Robot. Comput.-Integr. Manuf..

[4] Liu C., Vengayil H., Lu Y., Xu X. (2019). A Cyber-Physical Machine Tools Platform using OPC UA and MTConnect. J. Manuf. Syst..

[5] Zhang L., Yu H., Wang C., Hu Y., He W., Yu D. (2024). A digital solution for CPS-based machining path optimization for CNC systems. J. Intell. Manuf..

[6] Zhang M., Tao F., Huang B., Liu A., Wang L., Anwer N., Nee A.Y.C. (2021). Digital twin data: Methods and key technologies. Digit. Twin.

[7] Li Y., Peng S., Li Y., Jiang W. (2020). A review of condition-based maintenance: Its prognostic and operational aspects. Front. Eng. Manag..

[8] Shen B., Chen B., Zhao C., Chen F., Xiao W., Xiao N. (2021). Review on the Research of Deep Learning in Mechanical Equipment Fault Prognostics and Health Management. Mach. Tool Hydraul..

[9] Wang C., Wang C., Wang K. (2021). Technology Research and Standard Development of Predictive Maintenance for Intelligent Manufacturing Equipment. China Stand..

[10] Sanchez H., Escobet T., Puig V. (2020). Health-aware Model Predictive Control of Wind Turbines using Stifness Degradation Approach. IFAC-PapersOnLine.

[11] Lu S., Gao Z., Xu Q., Jiang C., Xie T., Zhang A. (2024). Remaining Useful Life Prediction Via Interactive Attention-Based Deep Spatio-Temporal Network Fusing Multisource Information. IEEE Trans. Ind. Electron..

[12] Peres F.A.P., Peres T.N., Fogliatto F.S., Anzanello M.J. (2019). Fault detection in batch processes through variable selection integrated to multiway principal component analysis. J. Process Control.

[13] Zhong X., Song X., Liu G., Zhao W., Fan W. (2024). A Data-Driven Method for Remaining Useful Life Prediction of Rolling Bearings Under Different Working Conditions. IEEE Trans. Reliab..

[14] Mandelli D., Wang C., Agarwal V., Lin L., Manjunatha K.A. (2024). Reliability modeling in a predictive maintenance context: A margin-based approach. Reliab. Eng. Syst. Saf..

[15] Cinus M., Confalonieri M., Barni A., Valente A. (2016). An ANN Based Decision Support System Fostering Production Plan Optimization Through Preventive Maintenance Management.

[16] Yan B., Sun Q., Shen L., Ma X. (2025). A Physical-Statistical Framework on Complex Mechanical System Fault Isolation. IEEE Trans. Reliab..

[17] Christen T., Macedo F. (2024). Theory of Fokker–Planck Equations for Reliability and Remaining Useful Lifetime Prognostics. IEEE Trans. Reliab..

[18] Yıldız G.B., Soylu B. (2023). Integrating preventive and predictive maintenance policies with system dynamics: A decision table approach. Adv. Eng. Inform..

[19] Alkabaa A.S., Taylan O., Guloglu B., Baik S., Sharma V., Mishra R., Alharbi R., Upreti G. (2024). A fuzzy ANP-based criticality analyses approach of reliability-centered maintenance for CNC lathe machine components. J. Radiat. Res. Appl. Sci..

[20] Zhong D., Xia Z., Zhu Y., Duan J. (2023). Overview of predictive maintenance based on digital twin technology. Heliyon.

[21] Tao F., Zhang M., Liu Y., Nee A.Y.C. (2018). Digital twin driven prognostics and health management for complex equipment. CIRP Ann..

[22] Deebak B.D., Al-Turjman F. (2021). Digital-twin assisted: Fault diagnosis using deep transfer learning for machining tool condition. Int. J. Intell. Syst..

[23] Luo W., Hu T., Ye Y., Zhang C., Wei Y. (2020). A hybrid predictive maintenance approach for CNC machine tool driven by Digital Twin. Robot. Comput.-Integr. Manuf..

[24] Yu J., Song Y., Tang D., Dai J. (2021). A Digital Twin approach based on nonparametric Bayesian network for complex system health monitoring. J. Manuf. Syst..

[25] Werner A., Zimmermann N., Lentes J. (2019). Approach for a holistic predictive maintenance strategy by incorporating a digital twin. Procedia Manuf..

[26] Booyse W., Wilke D.N., Heyns S. (2020). Deep digital twins for detection, diagnostics and prognostics. Mech. Syst. Signal Process..

[27] Predix Platform. GE Digital. https://www.cctech.co.in/platforms/ge-predix.

[28] Siemens Digital Twin. https://www.plm.automation.siemens.com/global/en/our-story/glossary/digital-twin/24465.

[29] Dai S., Zhao G., Yu Y., Zheng P., Bao Q., Wang W. (2021). Ontology-based information modeling method for digital twin creation of as-fabricated machining parts. Robot. Comput.-Integr. Manuf..

[30] Lu Y., Liu C., Kevin I., Wang K., Huang H., Xu X. (2019). Digital Twin-driven smart manufacturing: Connotation, reference model, applications and research issues. Robot. Comput.-Integr. Manuf..

[31] Yang X., Hu Y., Yu H., Wang C. (2021). Tool Wear Prediction Method Based on Residual Time Series Convolution Network. The 5th International Conference on Algorithms, Computing and Systems.

[32] Sun H., Zhang J., Mo R., Zhang X. (2020). In-process tool condition forecasting based on a deep learning method. Robot. Comput.-Integr. Manuf..

[33] Liu Z., Wang B., Li C., Yu M., Ding S. (2020). Fabric defect detection based on deep-feature and low-rank decomposition. J. Eng. Fibers Fabr..

[34] PHM Society 2010 PHM Society Conference Data Challenge. https://phmsociety.org/phm_competition/2010-phm-society-conference-data-challenge/.

[35] (2009). Mechanical Vibration—Evaluation of Machine Vibration by Measurements on Non-Rotating Parts.

[36] (2012). Test Code for Machine Tools.

